# The Effects of Lung Protective Ventilation or Hypercapnic Acidosis on Gas Exchange and Lung Injury in Surfactant Deficient Rabbits

**DOI:** 10.1371/journal.pone.0147807

**Published:** 2016-02-03

**Authors:** Helmut D. Hummler, Katharina Banke, Marla R. Wolfson, Giuseppe Buonocore, Michael Ebsen, Wolfgang Bernhard, Dimitrios Tsikas, Hans Fuchs

**Affiliations:** 1 Division of Neonatology and Pediatric Critical Care, Department of Pediatrics, Children’s Hospital, Ulm University, 89070 Ulm, Germany; 2 Departments of Physiology, Medicine and Pediatrics, *CENTRe*: Collaborative for Environmental and Neonatal Therapeutics Research; Temple Lung Center; Center for Inflammation, Translational and Clinical Lung Research, Temple University School of Medicine, Philadelphia, Pennsylvania, United States of America; 3 Pediatric Neonatology Unit, Department of Molecular and Developmental Medicine, University Hospital of Siena, Siena, Italy; 4 Institute for Pathology, Medizinisches Versorgungszentrum, Staedtisches Krankenhaus Kiel, Germany; 5 Department of Neonatology, Children’s Hospital, University of Tuebingen, Tuebingen, Germany; 6 Centre of Pharmacology and Toxicology, Hannover Medical School, Hannover, Germany; Icahn School of Medicine at Mount Sinai, ARGENTINA

## Abstract

**Background:**

Permissive hypercapnia has been shown to reduce lung injury in subjects with surfactant deficiency. Experimental studies suggest that hypercapnic acidosis by itself rather than decreased tidal volume may be a key protective factor.

**Objectives:**

To study the differential effects of a lung protective ventilatory strategy or hypercapnic acidosis on gas exchange, hemodynamics and lung injury in an animal model of surfactant deficiency.

**Methods:**

30 anesthetized, surfactant-depleted rabbits were mechanically ventilated (FiO_2_ = 0.8, PEEP = 7cmH_2_O) and randomized into three groups: Normoventilation-Normocapnia (NN)-group: tidal volume (Vt) = 7.5 ml/kg, target PaCO_2_ = 40 mmHg; Normoventilation-Hypercapnia (NH)-group: Vt = 7.5 ml/kg, target PaCO_2_ = 80 mmHg by increasing FiCO_2_; and a Hypoventilation-Hypercapnia (HH)-group: Vt = 4.5 ml/kg, target PaCO_2_ = 80 mmHg. Plasma lactate and interleukin (IL)-8 were measured every 2 h. Animals were sacrificed after 6 h to perform bronchoalveolar lavage (BAL), to measure lung wet-to-dry weight, lung tissue IL-8, and to obtain lung histology.

**Results:**

PaO_2_ was significantly higher in the HH-group compared to the NN-group (p<0.05), with values of the NH-group between the HH- and NN-groups. Other markers of lung injury (wet-dry-weight, BAL-Protein, histology-score, plasma-IL-8 and lung tissue IL-8) resulted in significantly lower values for the HH-group compared to the NN-group and trends for the NH-group towards lower values compared to the NN-group. Lactate was significantly lower in both hypercapnia groups compared to the NN-group.

**Conclusion:**

Whereas hypercapnic acidosis may have some beneficial effects, a significant effect on lung injury and systemic inflammatory response is dependent upon a lower tidal volume rather than resultant arterial CO_2_ tensions and pH alone.

## Introduction

Neonates with respiratory distress syndrome (RDS), and children and adults with acute respiratory distress syndrome (ARDS) often require supplemental oxygen and mechanical ventilation because of decreased arterial PO_2_, which by itself may induce further ventilator induced lung injury (VILI) and result in significant mortality and morbidity [1–3]. Current approaches to reduce VILI take into account the concept of “volutrauma” emphasizing the mechanical stress by shear forces as a key factor inducing further injury when an often heterogeneously injured lung with protein-rich fluid filled alveoli is exposed to mechanical in- and deflation with gas [1,3,4]. Reducing tidal volume may attenuate mechanical disruption of the alveolar-capillary barrier, but may result in hypercapnic acidosis, which often is referred to as “permissive hypercapnia”. Hypercapnia has been shown to improve ventilation-perfusion matching resulting in improved PaO_2_[5], which may allow reduction of ventilator settings and thus contribute to lung protection. Clinical data clearly suggests that a low tidal volume ventilatory strategy is lung-protective and may improve outcome [1,6,7]. Utilization of this “permissive hypercapnia” strategy in patients was associated with improved outcome in several clinical studies in adults with ARDS [6–8], but not in studies in preterm infants with RDS, both terminated early [9,10]. The traditional concept of reducing mechanical stress induced by shear forces via low tidal volume ventilation as the key factor of “permissive hypercapnia” to reduce VILI has recently been challenged. Experimental data on lung-protective effects of hypercapnic acidosis without reduction in tidal volume in models of ischemia-reperfusion injury [11], ventilator induced lung injury [12,13] and surfactant deficiency [14] have challenged the concept that the decrease in tidal volume is the main factor for lung protection. Furthermore, reducing tidal volume may be associated with impaired oxygenation secondary to increased intrapulmonary shunt [15]. These studies have demonstrated that hypercapnic acidosis, induced by adding CO_2_ into the inspiratory gas, attenuates lung injury compared to normocapnic control animals, although tidal volume was identical. However, there are no studies assessing the degree of lung injury in subjects with surfactant deficiency exposed to hypercapnia with a “normal” tidal volume proven to be lung protective in clinical studies [6], as compared to a strategy with a similar degree of hypercapnia using very small tidal volumes. Therefore, we compared the effects of a lung protective ventilator strategy using a very small tidal volume resulting in permissive hypercapnia to that of a normal tidal volume with hypercapnia induced by increasing FiCO_2_ and a ventilator strategy of a normal tidal volume and normocapnia on gas exchange, lung injury, and hemodynamics in an animal model of surfactant deficiency (ARDS). We hypothesized that hypercapnia improves PaO_2_ by attenuating the degree of lung injury during a hypoventilation strategy with very small tidal volumes only.

## Materials and Methods

### Animal preparation

All animals were cared for according to the current version of the German law on the protection of animals and in strict accordance with the recommendations in the Guide for the Care and Use of Laboratory Animals of the National Institutes of Health. The experiments were approved by the governmental animal care committee (Regierungspraesidium Tuebingen, Permit No. 721). A peripheral i.v. catheter (24G) was inserted in an ear vein to induce anesthesia. Thirty adult New Zealand White Rabbits (weight: 3393±129 g; [mean ± SD]) were given 0.5 mg atropine i.v., and were anesthetized with ketamine (13.7±2.9 mg/kg) and xylazine (1.4±0.3 mg/kg) i.v. All subsequent procedures were performed under anesthesia, and all efforts were made to minimize suffering. After supine positioning, the animals were intubated with a 3.5 mm internal diameter cuffed endotracheal tube. A rectal temperature probe (Siemens Sirecust 302, Erlangen, Germany) was placed and a core temperature of 38.5–39.5°C was maintained using a heating mattress and an overhead warmer (Babytherm 8000, Dräger, Lübeck, Germany). The animals were placed on volume-controlled, positive pressure ventilation using a Stephanie infant ventilator (Fritz Stephan Medizintechnik GmbH, Gackenbach, Germany) with heated and humidified gas and the following settings: FiO_2_: 0.4, tidal volume: 7.5 ml/kg, PEEP: 3 cm H_2_O, inspiratory time: 0.5 s, ventilator rate: 40/min. The ventilator rate was adjusted to maintain a PaCO_2_ within the target range of 35–45 mmHg. The inspiratory time was decreased to 0.4 s if a ventilator rate beyond 60 inflations/min was necessary to ensure that expiratory was always longer than inspiratory time,. Anesthesia was maintained by a continuous infusion of ketamine (67.6±11.0 mg/kg/h) and xylazine (6.8±1.1 mg/kg/h), and the dose was adjusted individually to maintain anesthesia deep enough to prevent spontaneous movements. Vecuronium (0.15 mg/kg i.v.) was given intermittently for muscle paralysis to prevent spontaneous respiratory activity as pilot experiments have shown that hypercapnic exposure may increase respiratory drive of the experimental animals, which would impair tidal volume targeting. Dextrose 2.5% with 135 mmol/l Na, 9 mmol/l K and 1 U Heparin/ml was administered at 5 ml/kg/h into a peripheral ear vein. A 3.5F arterial femoral line was inserted for continuous blood pressure monitoring and sampling for blood gas analyses. It was continuously perfused with heparinized (1 U/ml) normal saline at a rate of 2 ml/h. A 4F thermodilution catheter was introduced via the right jugular vein. Its tip was successfully placed into the pulmonary artery in 16 out of the 30 animals to measure cardiac output by thermodilution with a Sat-2 Cardiac Output Monitor (Baxter, Santa Ana, CA). The pulmonary arterial and central venous lines were continuously perfused with heparinized normal saline (1 U/ml) at a rate of 2 ml/h. Esophageal pressure was measured using a fluid-filled 5F feeding tube with its tip placed into the distal esophagus [16]. This tube was perfused continuously with water (3 ml/h) to avoid bubble formation. Airway, esophageal, arterial, central venous and pulmonary arterial blood pressures were measured with Sorenson Transpac pressure transducers (Transpac 4, Abbott Critical Care Systems, North Chicago, IL) connected to amplifiers (Gould, Valley View, OH). All pressure transducers were calibrated using a water or mercury manometer. Immediately before data acquisition, correct placement of the esophageal tube was checked by performing airway occlusions and by comparing airway and esophageal pressure [16]. A ratio of phasic esophageal and airway pressure changes of 1.0±0.1 was accepted. The airflow signal was derived from the ventilator, calibrated by a mass flow meter (Gas Products Model 8270, Matheson, Montgomeryville, PA). All signals were digitized at a frequency of 100 Hz and recorded simultaneously using a computerized data acquisition system (DATAQ Instruments, Inc., Akron, OH).

### Experimental protocol

After instrumentation FiO_2_ was increased to 0.8, baseline measurements before lung lavage were recorded, and surfactant deficiency was induced by repeated lung lavages as described previously [17]. In brief, lungs were lavaged with 15 ml/kg warmed normal saline every 10 min to aim for a PaO_2_ <200 mmHg for at least 15 min. Baseline measurements after lavage were recorded thereafter (time 0 h). Volume-controlled ventilation was continued with the settings described above, except for the positive end-expiratory pressure (PEEP) which was increased to 7 cmH_2_O, and animals were randomized into three groups using sealed envelopes: Group 1 was called the Normoventilation-Normocapnia (NN)-group: Vt was 7.5 ml/kg, and the ventilator rate was adjusted to maintain PaCO_2_ within the target range of 35–45 mmHg. Group 2 was called the Normoventilation-Hypercapnia (NH)-group: Vt was 7.5 ml/kg, and a target PaCO_2_ of 75–85 mmHg was achieved by adding approximately 4% CO_2_ into the inspiratory limb. Group 3 was called the Hypoventilation-Hypercapnia (HH)-group, the same target PaCO_2_ of 75–85 mmHg was achieved by lowering the tidal volume to 4.5 ml/kg and the ventilator rate if necessary.

### Hemodynamic and other support

Hemodynamic support was given by an intravenous volume infusion of 15 ml/kg normal saline given i.v. within 15 min, whenever the diastolic blood pressure dropped to <40 mmHg, and by continuous dopamine infusion in increments of 5 μg/kg/min up to 30 μg/kg/min, whenever two volume infusions were unsuccessful. Whenever the base deficit exceeded 10 mmol/l, the difference between the actual base deficit and 5mmol/l was corrected with sodium bicarbonate 4.2% i.v. within 15 min using the formula: mEqual NaHCO_3_ = (actual base deficit– 5 mmol/l) x 0.3 x kg. Animals surviving the 6 h observational period were sacrificed after 6 h with a thiopental overdose (50 mg i.v.).

### Physiological variables

Arterial, central venous, and pulmonary arterial blood pressure, and heart rate were obtained as average values from the last 5 min of each 30 min interval. Cardiac output was measured hourly and the average of three serial thermodilution measurements at each time point was calculated and corrected for weight. Peak inspiratory pressure, mean airway pressure, minute ventilation, tidal volume, respiratory rate and lung compliance were measured in 10 randomly selected breaths during the same 5 min period and was calculated using a computer program based on the equation of motion as described before [18].

### Postmortem protocol

Immediately after death, the chest was opened, the lungs were inflated using a continuous airway pressure of 20 cmH_2_O for 1 min, and then left at 10 cmH_2_O. A cannula was placed into the pulmonary artery, to perfuse the lung in situ with 100 ml Ringer’s lactate containing procaine (250 mg/l), heparin (20 U/ml), and CaCl (2,2 mmol/l) with a perfusion pressure of 30 cmH_2_O with the left atrium opened. Thereafter, the right lung was excised and its wet weight was measured immediately. Thereafter, the right lung was lavaged twice with 7.5 ml/kg normal saline. Small samples (0.5 g) of dependent and non-dependent areas of the right lung were taken, weighed and homogenized in 4 ml normal saline to measure tissue concentrations of IL-8. The remainder of the right lung was dried in a drying oven to calculate the wet-to-dry weight ratio after daily weight measurements did not change for three days, taking into account the tissue lost for tissue samples. The left lung was perfused with a formaldehyde (3.6%)/glutaraldehyde (0.5%) solution for 10 min. Finally the left lung was removed and submersed in the same solution maintaining an airway pressure of 10 cmH_2_O for at least 8 h.

### Blood and tissue sampling, and BAL protocol

Arterial blood samples were drawn every 30 mins and immediately processed using an Omni 9 Analyzer (Roche Diagnostics, Mannheim) to measure blood gases, arterial oxygen saturation of hemoglobin as measured by CO-oximetry and lactate. Further, blood samples were drawn in Li-heparin tubes every 2h, centrifuged immediately to store plasma at –80°C for measurements of interleukin-8 (IL-8), and at 6h to measure total 15(*S*)-8-*iso*-prostaglandin F_2α_ (15(*S*)-8-*iso*-PGF_2α_), advanced oxidation protein products (AOPP), total hydroperoxydes (TH) and non-protein bound iron (NPBI).

All effluent aliquots of the initial bronchoalveolar lavage (BAL) to induce lung injury were pooled, centrifuged (200xg for 10 min at 4°C) to remove cells. The supernatant was stored at –80°C for further analysis of total protein and IL-8. The BAL fluid (BALF) obtained postmortem from the right lung was processed identically. Values were corrected for volume differences of the BAL volume recovered.

### Biochemical analyses

Interleukin-8: Lung tissue (0.2–0.5 g) was washed twice with phosphate-buffered saline and homogenized in 5 ml of the buffer solution. The homogenate was centrifuged (5000xg for 5 min at 4°C) to obtain supernatant for measurement of IL-8 concentration. Lung tissue supernatant, plasma and BAL fluid supernatant, was determined using a commercial rabbit-specific ELISA kit (OptEIA rabbit IL-8 set, PharMingen, San Diego, CA). The limit of detection was 3.1 pg/ml with inter-assay and intra-assay coefficient of variation of 8.4 and <5%, respectively.

Total (i.e. free and esterified) 15(*S*)-8-*iso*-PGF_2α_ was measured in 500-μL plasma samples by GC-MS/MS after saponification and immunoaffinity column chromatography extraction and derivatization as described elsewhere [19]. [3,3´,4,4´-2H_4_]15(S)-8-iso-PGF2α was used as the internal standard at a concentration of 100 pg/mL plasma.

Measurements of AOPP, TH and NPBI: Blood samples were immediately centrifuged at 5000 rpm and the supernatants were stored at −80°C after having added butylated hydroxytoluene (BHT) to prevent oxidation during processing. NPBI was determined according to previously described methods [20]. AOPP were measured as described by Witko-Sarsat et al. [21] using spectrophotometry on a microplate reader and expressed as μmol/l chloramine-T equivalents. TH production was measured with a d-ROMs Kit (Diacron s.r.l., Grosseto, Italy). This method makes it possible to estimate the total amount of hydroperoxide present in a blood sample by using a spectrophotometric procedure [22].

Total phospholipids were quantified from surfactant pellets of initial and final BALF. For this, cell-free BALF was centrifuged at 60,000xg for 60 min at 4°C. The pellets were re-suspended with 0.5 ml buffered Ringer’s solution with 1.5 mmol/l CaCl_2_ and frozen at -80°C until analysis.

### Lung histology

Fixed tissue samples were obtained from dependent and nondependent parts of the upper and lower lobes of the left lung in each animal. Samples were processed for light microscopy and slides were stained with hematoxylin/eosin, and evaluated by a pathologist (M.E.) blinded to the animal’s group assignment by using a previously described score [23]. Briefly, variables scored were atelectasis, alveolar and interstitial inflammation, alveolar and interstitial edema, necrosis, and overdistension. Each variable was scored by using a 0–4 point scale with no injury = 0, injury in 25, 50, 75% and throughout the field scoring 1,2,3, and 4.

### Statistical analysis

Differences of categorical variables between groups were analyzed by using Fisher’s exact test and of continuous variables by 2-tailed *t*- tests, Wilcoxon rank sum tests, ANOVA, ANOVA for repeated measures, Kruskal-Wallis ANOVA, Kruskal-Wallis ANOVA for repeated measures where appropriate. Missing data points of repeated measurements secondary to early deaths were handled using a general linear model (Sigmastat V.2.03, Systat Software Inc., San Jose, CA). A p<0.05 was considered significant. The primary outcome measure was PaO_2_. A pre-study sample size calculation revealed that 9 animals/group would be necessary to detect a difference of 80 mmHg in PaO_2_ between groups assuming an α = 0.05, and a β = 0.2, and a standard deviation in PaO_2_ measurements of 51 mmHg, which was estimated from a previous study using the same animal model [24]. To compensate for potential data loss the sample size was increased to 10 animals/group. Values are presented as mean ± SD if normally distributed, or as median (range) unless otherwise stated.

## Results

**Fig 1:**
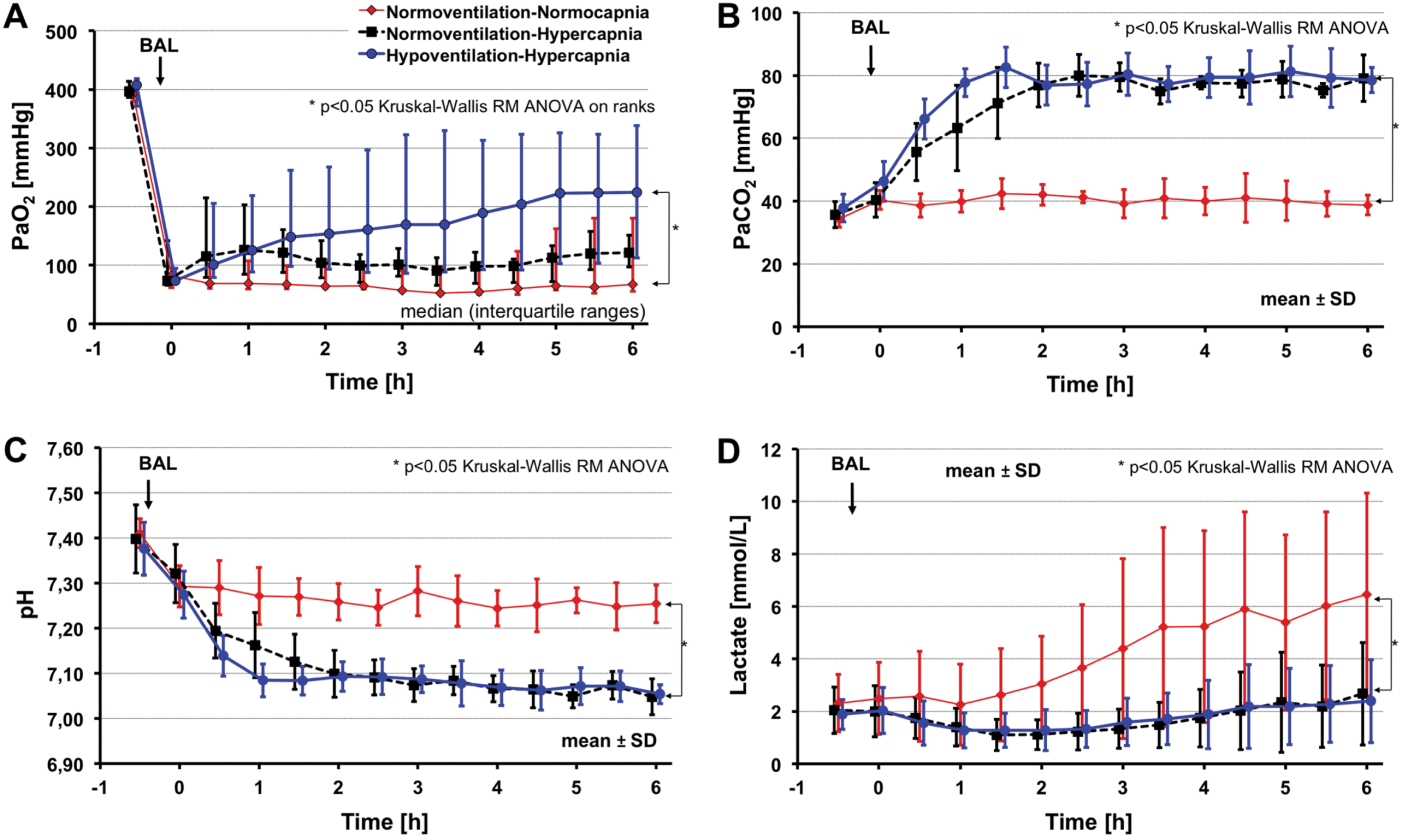
Gas exchange (PaO_2_ [A], PaCO_2_ [B]) and acid base characteristics (pH [C], lactate [D]) over experimental time. Time = 0 refers to time of randomization. BAL = bronchoalveolar lavage. PaO_2_ was significantly higher in the HH- vs. the NN-group. Arterial pH and lactate were lower in both hypercapnia groups as compared to the NN-group; * = p< 0.05.

Of the 30 animals, 27 survived to the end of the experimental period. Two animals of the NN-, and one animal of the NH-group died prematurely due to cardiovascular failure. Fig 1 shows gas exchange, pH and lactate levels over time. PaO_2_ was significantly higher in the HH- vs. the NN-group. PaCO_2_ was maintained close to the respective targets in all 3 groups. Arterial pH and lactate were lower in both hypercapneic groups as compared to the NN-group (p<0.05).

**Fig 2:**
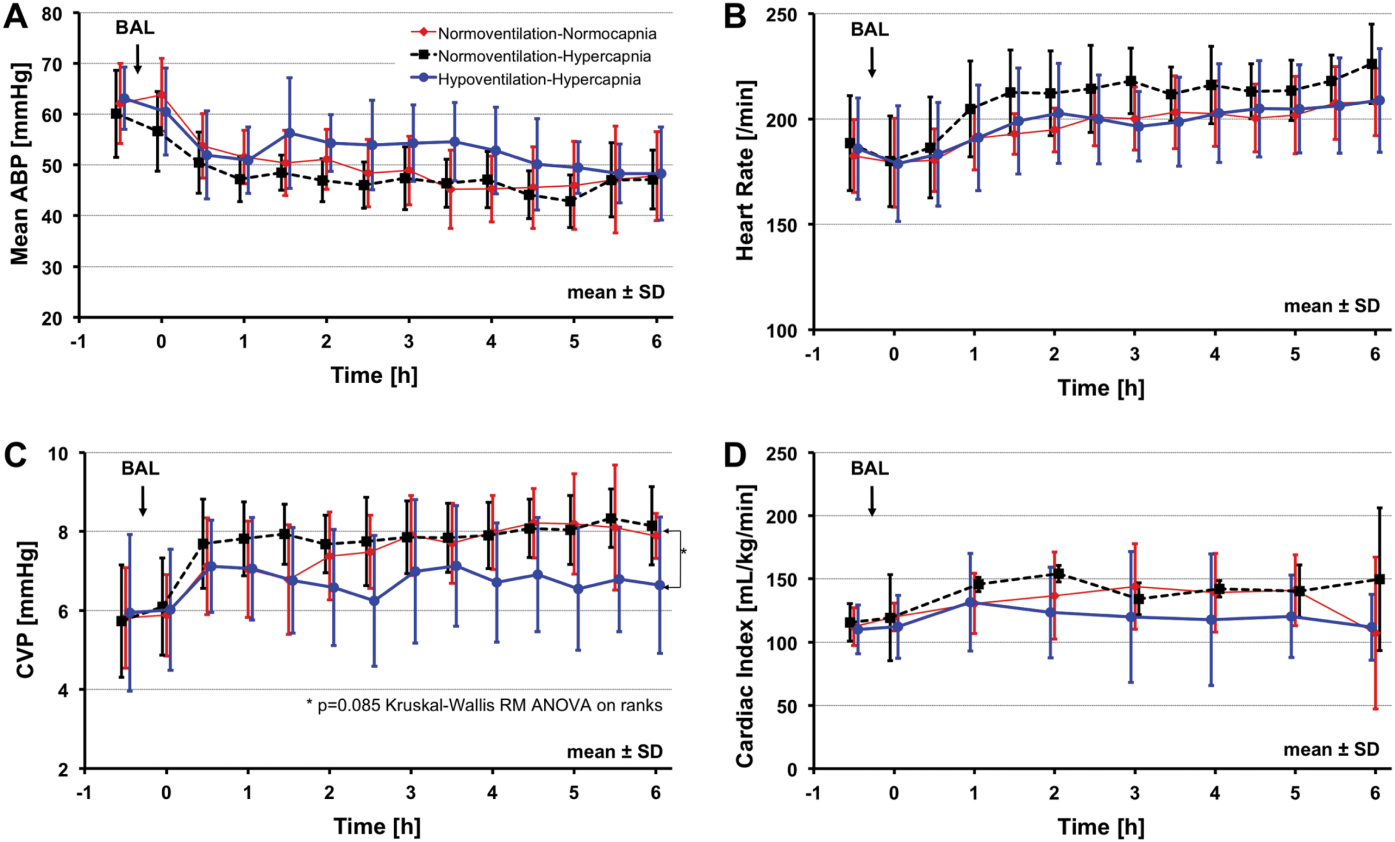
Hemodynamic parameters over time: Arterial blood pressure [A], heart rate [B], central venous pressure [C] and cardiac index [D] over experimental time. Time = 0 refers to time of randomization. BAL = bronchoalveolar lavage. There were no significant differences between groups.

Mean arterial blood pressure, heart rate and cardiac output were not significantly different comparing the 3 groups (Fig 2). There was no significant difference in pulmonary arterial pressure (data not shown), but there was a trend towards a lower central venous pressure in the HH-group as compared to the 2 normoventilation groups (NN and NH). Interventions according to the protocol to stabilize blood pressure were less likely to be necessary in the HH-group (Table 1). Moreover, less volume replacement was needed and the average dopamine dose was lower in HH-group as compared to the both normoventilation groups reaching statistical significance compared to the NH-group. Furthermore, less NaHCO_3_-replacement was necessary in the HH-group according as compared to the NN group.

**Table 1:** Interventions to stabilize hemodynamics and pH.

	Normoventilation-Normocapnia	Normoventilation-Hypercapnia	Hypoventilation-Hypercapnia
Dopamine dose [μg/kg/min] ^a^	5.6 (0–15.9) ^‡^	15.1 (1.3–23.8)	1.9 (0–17.7) ^†^
Volume replacement [ml/kg] ^b^	48.3 (9.2–88.2) ^‡^	83.6 (8.2–122.0)	30.1 (8.7–83.9) ^†^
NaHCO_3_ [mEqu/kg] ^b^	25 (0–45)	15 (0–32)	14 (0–22) *

^a^ = averaged across time;

^b^ = cumulative amount given;

* = p < 0.05 (HH-group vs. NN-group);

^†^ = p<0.05 (HH-group vs. NH-group);

^‡^ = p < 0.05 (NN-group vs. NH-group).

Minute ventilation, tidal volume, peak inspiratory and mean airway pressure was lower in the HH-group as expected by the protocol (Table 2). There was no significant difference in lung compliance between groups (Table 2).

**Table 2:** Ventilation parameters and pulmonary mechanics.

	Normoventilation-Normocapnia	Normoventilation-Hypercapnia	Hypoventilation-Hypercapnia
Tidal Volume [ml/kg]	7.4 ± 0.2	7.3 ± 0.3	4.5 ± 0.3 *
Ventilator Rate [breaths/min]	40.0 ± 8.2	40.8 ± 4.4	32.8 ± 8.5 *
Minute Ventilation [ml/kg/min]	314 ± 75	306 ± 37	158 ± 45 *
Peak Inspiratory Pressure [cmH_2_O]	25.7 ± 3.3	22.3 ± 3.3	18.2 ± 4.0 *
Mean Airway Pressure [cmH_2_O]	11.7 ± 1.4	10.8 ± 1.0	9.5 ± 1.2 *
Lung Compliance [ml/kg/cmH_2_O]	0.42 ± 0.09	0.51 ± 0.13	0.40 ± 0.12

Values are mean ± SD;

* = p < 0.05 (HH-group vs. NN-group).

**Fig 3:**
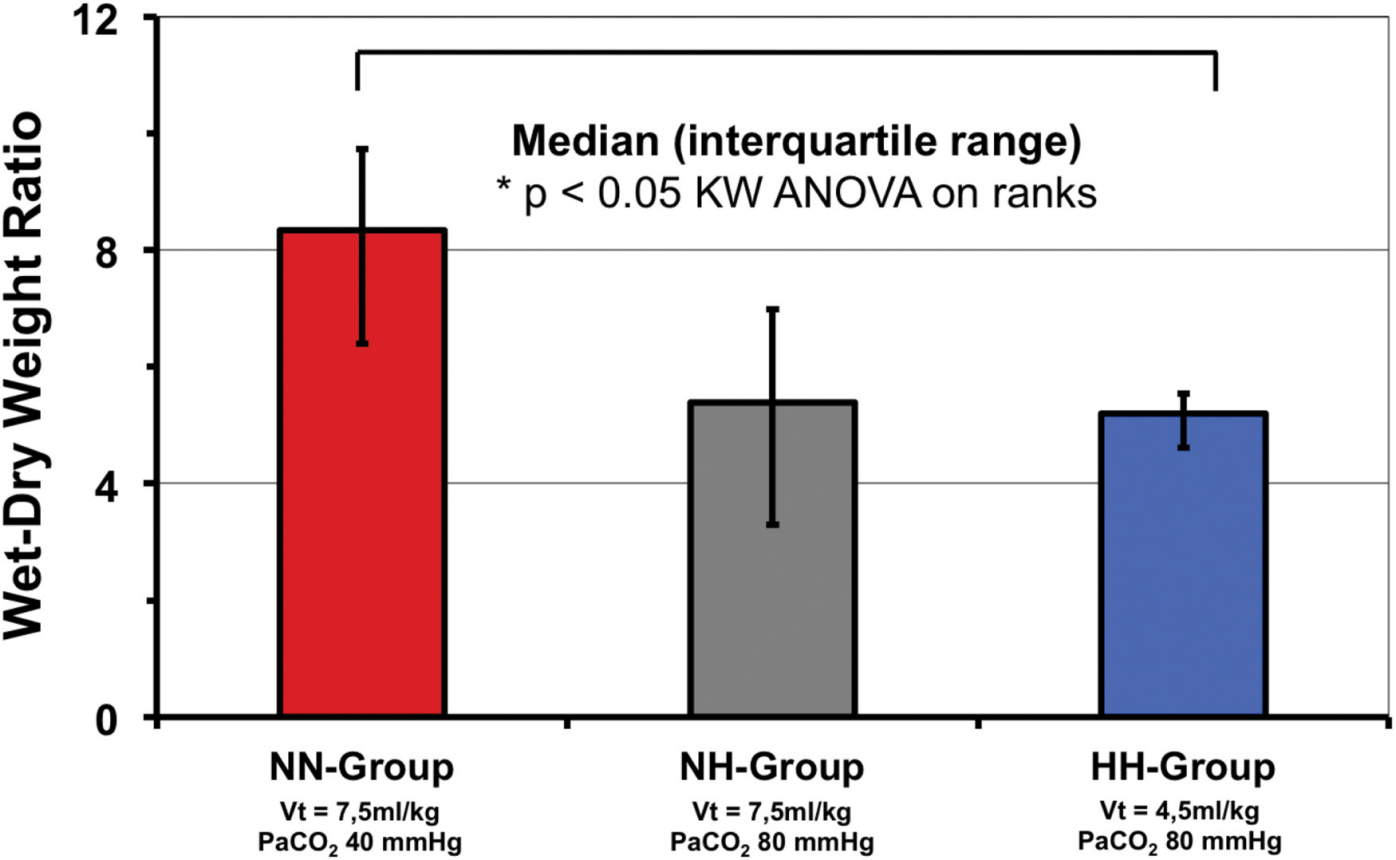
Wet-dry-weight ratio of excised lungs. * = p< 0.05. NN vs. NH groups: p = 0.07.

Wet-to-dry ratio of excised lungs tended to be lower in the NH group (p = 0.07) and was significantly lower in the HH-group as compared to the NN-group (p<0.05; Fig 3). Lung histology is shown in Table 3. Scores were significantly higher comparing lower lobes vs. upper lobes and for dependent vs. non-dependent specimen for most scores (data not shown). Scores for atelectasis, alveolar inflammation, alveolar hemorrhage, alveolar edema and the sum score were significantly lower for the HH-group as compared to the NN-group indicating less lung injury. In some scores evaluated the NH-group showed trends towards lower scores compared to the NN-group, none reaching statistical significance (Table 3).

**Table 3:** Lung histology scores.

	Normoventilation-Normocapnia (NN-group)	Normoventilation-Hypercapnia (NH-group)	Hypoventilation-Hypercapnia (HH-group)
Atelectasis	0.8 (0–1.3)	0.3 (0–1.3) ^a^	0 (0–1.0) **
Alveolar inflammation	1.5 (1.3–2.0)	1.8 (0–2.5)	0.3 (0–2.5) *^,^^b^
Interstitial inflammation	1.8 (1.3–2.5)	1.8 (0–2.5)	1.0 (0–2.5)
Alveolar hemorrhage	0.8 (0.5–1.0)	0.8 (0–1.0)	0.1 (0–1.0) **^,^^†^
Interstitial hemorrhage	0.9 (0.8–1.5)	0.8 (0–1.5)	0.4 (0–1.5) ^c^
Alveolar edema	0.8 (0.3–1.8)	0.3 (0–1.0)	0 (0–0.8) **
Interstitial edema	0.6 (0–0.8)	0.8 (0–1.3)	0.3 (0–0.8) ^†^
Necrosis	0	0	0
Overdistension	2.6 (2.3–3.0)	2.8 (2.5–3.0)	3.0 (2.8–3.0) *
**Sum-Score**	**9.9 (7.3–12.5)**	**9.3 (3.5–11.5)**	**4.8 (3.3–11.8)** *

Values are median (minimum–maximum);

* = p < 0.05 and

** = p<0.01 (HH-group vs. NN-group);

^†^ = p<0.05 (HH-group vs. NH-group);

^a^ = p = 0.09 (NH-group vs. NN-group);

^b^ = p = 0.08 (HH-group vs. NH-group);

^c^ = p = 0.09 (HH-group vs. NN-group); (ANOVA or Kruskal Wallis ANOVA on ranks where appropriate).

**Fig 4:**
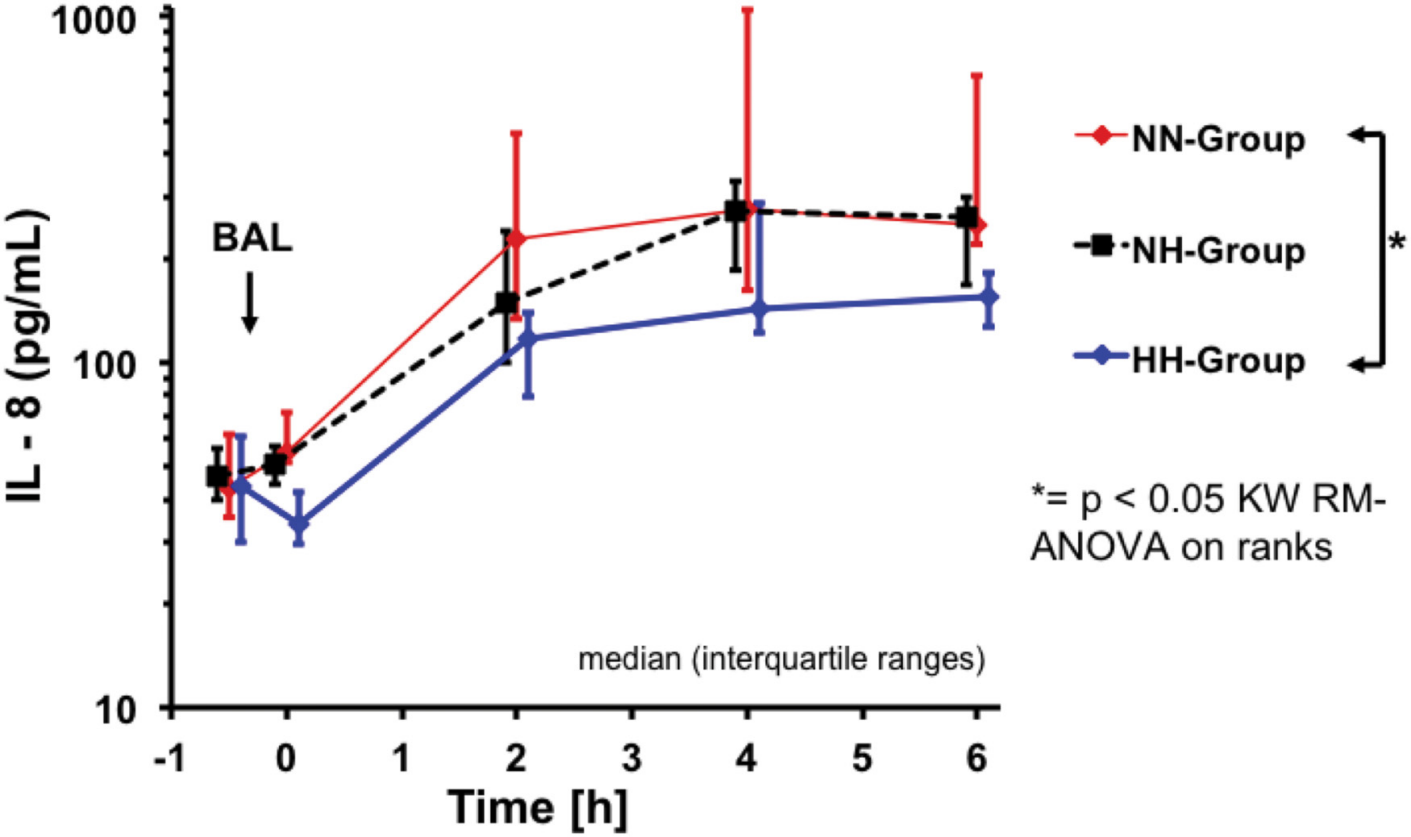
Plasma interleukin-8 over time. * = p< 0.05, NN- vs. HH-groups.

Total protein concentration in BALF was lower in the NN-group compared with the HH-group only (Table 4). IL-8 in BALF was not significantly different between groups, whereas lung tissue IL-8 concentrations were significantly lower in the HH-group as compared to the NN-group in dependent lung tissue specimen (Table 4). Plasma Interleukin-8 was lower in the HH-group as compared to both normoventilation groups reaching statistical significance comparing HH and NN-groups (Fig 4; p<0.05).

**Table 4:** Protein and IL-8 in broncheal alveolar lavage fluid and lung tissue IL-8.

	Normoventilation-Normocapnia (NN-group)	Normoventilation-Hypercapnia (NH-group)	Hypoventilation-Hypercapnia (HH-group)
BALF Protein [mg/kg]	24.3 (5.0–56.4)	16.1 (3.4–35.9)	7.2 (2.6–49.6) *
BALF IL-8 [ng/kg]	29.8 (8.1–188.4)	18.3 (4.5–64.6)	14.9 (1.7–61.9)
Lung Tissue IL-8dependent lung [pg/g]	23.2 (6.4–100.7)	23.9 (2.0–55.8)	7.5 (3.9–19.9) * †
Lung Tissue IL-8non-dependent lung [pg/g]	17.7 (5.1–232.9)	42.3 (3.6–112.0)	12.6 (0.6–32.9)

Values are median (minimum–maximum); BALF IL-8 and lung tissue IL-8 were measured in right-sided lungs. Lung tissue IL-8 levels are normalized for tissue weight.

* = p < 0.05 (HH-group vs. NN-group);

^†^ = p<0.05 (HH-group vs. NH-group).

Phospholipid content in 60,000xg pellets of BALF was decreased at the end of the experiment compared with the initial values in all 3 groups, independent of ventilation strategy (Table 5).

**Table 5:** Broncheal alveolar lavage: Surfactant (phospholipid) content.

	Normoventilation-Normocapnia (NN-group)	Normoventilation-Hypercapnia (NH-group)	Hypoventilation-Hypercapnia (HH-group)
Initial BAL [mg/kg]	298 (121–506)	299 (231–467)	305 (249–829)
Final BAL [mg/kg]	49 (30–168)	58 (35–162)	47 (31–169)

Values are median (minimum–maximum). Surfactant phospholipid content was analyzed from BAL obtained from both lungs initially and from final BAL of the right lung. Values were corrected for BAL volume. No significant difference between groups was detected.

There was a significant difference in 15(*S*)-8-*iso*-PGF_2α_ levels between NN- and HH-groups at baseline before lavage, but there was no difference in 15(*S*)-8-*iso*-PGF_2α_ levels between all experimental groups after 6h (Table 6). Whereas there were no differences in TH levels between all experimental groups at the end of the experiment, AOPP levels were significantly lower in the HH-group as compared to the normocapnia group and non-protein bound iron levels were significantly lower in both hypercapnia groups (Table 6).

**Table 6:** Markers of oxygen toxicity. 15(*S*)-8-*iso*-PGF_2α_ levels (measured at baseline and at the end of the experiment after 6h), Advanced Oxidation Protein Products (AOPP) and Total Hydroperoxydes (TH) and Non-protein Bound Iron (NPBI), measured at the end of the experiment (6h) in plasma samples.

	Normoventilation-Normocapnia	Normoventilation-Hypercapnia	Hypoventilation-Hypercapnia
15(*S*)-8-*iso*-PGF_2α_ [pg/mL]			
Baseline before lavage	59.8 ± 15.2	44.4 ± 18.9	46.1 ± 9.9 *
End of experiment	51.2 ± 19.3	60.5 ± 24.7	57.3 ± 24.1
AOPP [μg/dL]	16.5 (4.1–42.3)	10.9 (4.1–23.4)	6.4 (1.5–18.5) *
TH [UCARR]	108 (65–162)	144 (68–214)	82 (29–193)
NPBI [mmol/L]	30.1 (16.2–81.7) ^‡^	3.8 (0.6–9.9)	3.3 (1.9–6.0)

Values are mean ± SD or median (range);

* = p < 0.05 (HH-group vs. NN-group);

^‡^ = p <0.05 (NN-group vs. NH- and HH-groups).

## Discussion

The aim of this study was to evaluate the effects of hypercapnic acidosis on the degree of lung injury in this animal model of surfactant deficiency. The design chosen for this study allows separating the protective effects by elevated PCO_2_ levels from the lung protective effects of a small tidal volume. Although there seems to be some effect of hypercapnic acidosis on PaO_2_, the hypothesis that hypercapnic acidosis improves PaO_2_ was proven for the combined effect of a small tidal volume and hypercapnia only. Furthermore, PaO_2_ was increased in the HH-group despite a lower peak and mean airway pressure as compared to both other groups. Lung injury was decreased in these animals as judged by wet-to-dry weight, BAL-Protein, histology. However, we could not prove similar protective effects in the animals of the NH-group. Furthermore, systemic IL-8 levels were significantly lower in the HH-group only, suggesting that systemic inflammation is reduced during low tidal volume use only. This finding suggests more loss of alveolar and systemic compartmentalization in NN- and NH-groups which has been described in animals exposed to injurious mechanical ventilation [25].

Differences between our findings and those of previous studies may be related to several factors. For example, the PEEP chosen in our study may have been sufficient to counteract the potential negative effects of decreased peak or mean airway pressure on pulmonary shunt and gas exchange. Arterial pO_2_ increased in our model very early after randomization in both hypercapnia groups, and the mechanism involved may be related to improved ventilation-perfusion matching secondary to pulmonary vasoconstriction and improved perfusion of non-dependent and better aerated lung areas [5]. Although data on PaO_2_, ventilator pressure, lung compliance, wet-to-dry weight, and BALF protein show trends towards protective effects in the NH-group as compared to the NN-group, a reduced tidal volume clearly was more protective than hypercapnia alone in our model. The lack of lung protection in our NH-group is in line with data from Rai et al. who did not detect a lung-protective effect of an elevated PaCO_2_ (by increasing FiCO_2_) in rabbits with surfactant deficiency during both, injurious (tidal volume 12 ml/kg, no PEEP) or lung-protective ventilation (tidal volume 5 ml/kg, PEEP 12.5 cmH_2_O) [26]. However, in contrast to Rai et al. [26] we aimed to minimize VILI by reducing tidal volume and ventilator rate to very low settings to implement permissive hypercapnia and we were able to show a protective effect of hypercapnia induced with a small tidal volume.

Hypercapnic acidosis has been shown to suppress nuclear factor-kappa B activation and production of IL-8 in experimental settings [27,28], and the use of a lower tidal volume in patients undergoing mechanical ventilation during surgery resulted in lower serum IL-8 levels [29]. We found lower IL-8 values in the dependent lung tissue samples and a trend towards lower values in the BALF, which may reflect anti-inflammatory properties of lower tidal volume and/or hypercapnic acidosis modulating lung injury. Our study design, however, does not allow to clarify if the lower tidal volume or hypercapnic acidosis, or both, decreased inflammation.

Phospholipid content of BALF, as a surrogate parameter for total surfactant in the alveolar space at the end of the experiment was not different between groups. This suggests that type II pneumocytes only partially compensated for the loss of surfactant, in spite of the residual pools within their lamellar bodies, and that there was no difference in the effect of ventilation parameters on surfactant homeostasis. As surfactant is partially lost along the airways during alveolar collapse and tidal volume affects surfactant turnover, the kinetics of surfactant secretion and re-uptake may nevertheless be different between the groups, which only can be addressed by isotope labeling [30]. Moreover, according to the Young-Laplace-equation alveolar collapse mostly occurs at low alveolar diameter during the end of the ventilation cycle, which is prevented by high PEEP. Therefore, the relatively high PEEP of 7 cmH_2_O may have prevented intrapulmonary shunt flow and substantially reduced the need of surfactant. This was previously shown in neonatal rats, where an autogenous PEEP by high respiratory rate is associated with very low surfactant values [30,31]. Together, all this may have contributed to the good oxygenation of these surfactant-deficient animals, highlighting that PEEP and tidal volume are at least as important for lung protection as surfactant homeostasis.

Markers on oxidative injury showed inconclusive results. Whereas we were able to document a lower NPBI in both hypercapnia groups and AOPP after 6h, the other markers and 15(*S*)-8-*iso*-PGF_2α_ levels at 6h did not show differences between groups in our short-term model. Other authors have demonstrated that exposure to hypercapnia attenuated tyrosine nitration and histologic lung injury in rats exposed to 60% oxygen for 14d [32], suggesting that hypercapnia may mediate hyperoxic lung damage. One might speculate that the high FiO_2_ used in our study resulted in a significant hyperoxic stress, which may not be attenuated by hypercapnia. However, the timeframe of our experiment was probably too short to assess a protective effect of hypercapnia on oxidative lung damage detectable by measuring F_2_-isoprostanes. During hypoxic conditions exposure to 10% CO_2_ was found to reduce total 8-isoprostane lung levels and reduced vascular remodeling and pulmonary hypertension [33].

Hemodynamic variables did not seem to be impaired by hypercapnic acidosis in our animals. In fact, animals of the NH group needed more volume replacement and dopamine than the other groups, and animals ventilated with low tidal volume (HH-group) needed less hemodynamic support, suggesting that the lower tidal volume may be the main factor for reduced requirement of cardiovascular support. Other data suggests that beta-adrenergic activation may occur secondary to hypercapnic acidosis [34]. Lactate levels were lower in both hypercapnia groups which may be related to decreased mitochondrial respiratory activity [35] and downregulation of the production of organic acids during acidosis [36] which protects acid-base balance in the setting of increased acid production [37].

In a previous experiment we have studied the effects of different tidal volumes with associated different levels of permissive hypercapnia on lung injury and gas exchange using the same model and were able to show that a tidal volume below 4–5 ml/kg with a PaCO_2_ of >80 mmHg did not increase lung protection further [38]. The major limitation of that study and of the present study is the short-term nature of both experiments showing beneficial effects within the 6 h study frame. Lung cell repair may be impaired in the setting of hypercapnic acidosis as shown in an short-term experimental study [39], but the relevance of these effects on long-term recovery from acute lung injury is unknown. Although we used an animal model with surfactant deficiency, the animals used do not have immature lungs and therefore the applicability of our findings to lung injury in preterm subjects may be limited. Another limitation is the fact that we did not use a 4^th^ group with low tidal volume and normocapnia. However, a low tidal volume/normocapnia approach using a higher ventilator rate may have resulted in air trapping. It would be feasible using tracheal gas insufflation to wash out CO_2_. However, tracheal gas insufflation may cause airway epithelial injury as adequate humidification of bias flow into the trachea is difficult to achieve.

Currently it is unknown to what level permissive hypercapnia can be safely employed in preterm infants, children or adults with severe lung injury. Retrospective data in preterm infants suggests an association of an increased incidence of Bronchopulmonary Dysplasia in association with hypercapnia during the first week of life after adjustment for exposed baseline risk, severity of illness and mean airway pressure [40]. In most randomized clinical trials PCO_2_ values up to 55 mmHg were tolerated during the first days of life and neither proved relevant clinical benefits, nor were they associated with relevant side-effects when compared to control groups aiming for normocapnia [9,41,42]. In a recently published randomized trial necrotizing enterocolitis was more frequent in the permissive hypercapnia group [10]. Because experimental evidence suggests that exposure to hypercapnic acidosis may cause clinically relevant changes in cerebral blood flow and cerebral tissue oxygenation [43] and adverse effects on cerebral cortex enzyme activity and protein expression in the immature brain [44], follow-up studies in preterm infants exposed to hypercapnic acidosis are needed to assess long-term effects on the developing brain of premature infants.

## Conclusions

We conclude that in this model of surfactant deficiency hypercapnic acidosis combined with a low tidal volume improves arterial oxygenation and protects against inflammation and lung injury. However, these effects were not significant for hypercapnic acidosis with a normal tidal volume, showing that either low tidal volume alone or the combination of low tidal volume with permissive hypercapnia is essential for lung protection. More research is needed to study the interaction between tidal volume and CO_2_ and their effects on hemodynamics and other systemic effects. Furthermore, clinical trials using different target ranges of tidal volume and PaCO_2_ looking at side effects and including long-term follow-up of these patients need to be performed to assess whether this approach of protective ventilation with permissive hypercapnia can be recommended for routine clinical use.

## Supporting Information

S1 File Arrive checklist. (PDF)
